# ARHGAP11A Is a Novel Prognostic and Predictive Biomarker Correlated with Immunosuppressive Microenvironment in Clear Cell Renal Cell Carcinoma

**DOI:** 10.3390/ijms24097755

**Published:** 2023-04-24

**Authors:** Huihui Yang, Hongning Zhang, Liuxu Zhang, Paizigul Tusuphan, Junfang Zheng

**Affiliations:** 1 Beijing Key Laboratory of Cancer Invasion and Metastasis Research, Department of Biochemistry and Molecular Biology, School of Basic Medical Sciences, Capital Medical University, Beijing100069, China; 2 Department of Pharmacology, School of Basic Medical Sciences, Capital Medical University, Beijing100069, China

**Keywords:** *ARHGAP11A*, RhoGAP, prognosis, immune infiltrates, renal cell carcinoma, IGF2BP3

## Abstract

Clear cell renal cell carcinoma (ccRCC) is a highly immunogenic tumor and immune dysfunction is associated with ccRCC poor prognosis. The RhoGTPase-activating proteins (RhoGAPs) family was reported to affect ccRCC development, but its role in immunity and prognosis prediction for ccRCC remain unknown. In the current study, we found *ARHGAP11A* was the only independent risk factor among 33 RhoGAPs (hazard ratio [HR] 1.949, 95% confidence interval [CI] 1.364–2.785). High *ARHGAP11A* level was associated with shorter overall survival (OS, HR 2.040, 95% CI 1.646–3.417) and *ARHGAP11A* is a prognostic biomarker for ccRCC. *ARHGAP11A* knockdown suppressed renal cell carcinoma (RCC) cell proliferation, colony formation, and migration, suggesting the promoting role of *ARHGAP11A* on RCC development. Mechanistically, *ARHGAP11A* might contribute to the suppressive tumor immune microenvironment (TIME). High *ARHGAP11A* level was correlated with infiltration of immunosuppressive cells (including T helper 2 (Th2) cells, regulatory T (Treg) cells, myeloid derived suppressor cells (MDSC), and M2 macrophage cells), activation of immunosuppressive pathways (IL6-JAK-STAT3 signaling and IFNγ response), and expression of inhibitory immune checkpoints (ICs). *ARHGAP11A* could promote T cell exhaustion and induce immune escape. ccRCC patients with low *ARHGAP11A* level were more suitable for immune checkpoint inhibitors (ICIs) therapy, while those with high *ARHGAP11A* level might benefit from a combination of *ARHGAP11A* blockade and ICIs. In all, *ARHGAP11A* might serve as a novel prognostic marker, therapeutic target, and predictor in the clinical response to ICIs therapy for ccRCC.

## 1. Introduction

Renal cell carcinoma (RCC) is a highly lethal malignancy in the urinary system [1]. Clear cell RCC (ccRCC) accounts for approximately 80% to 90% of RCC [2]. Despite the presence of leukocyte infiltrates [3,4,5], a high degree of tumor-infiltrating lymphocytes (TILs) is associated with a poor prognosis in ccRCC [6,7]. One reason for the inability of TILs to mediate antitumor function is an immune suppressive microenvironment mediated by infiltration of regulatory T (Treg) cells and myeloid cell types. Another possibility is the dysfunction of effector T cells and the inhibition of antigen presenting cells via upregulation of suppressive factors such as immune checkpoint (IC) molecules [8,9]. Recently, immune checkpoint inhibitors (ICIs) and combination therapies have revolutionized the treatment of advanced ccRCC. However, some patients do not benefit from checkpoint blockade due to immune escape [10]. Therefore, it is crucial to enhance clinical efficacy of ICIs therapy by understanding the molecular mechanisms underlying ccRCC development, identifying predictive biomarkers that can predict the efficacy of ICIs therapy, and sensitizing patients who are resistant to ICIs therapy.

Abnormalities in Rho GTPase signaling are closely associated with cancer development [11,12]. The activation and inactivation of Rho GTPase signaling are regulated by guanine nucleotide exchange factors (RhoGEFs) and GTPase-activating proteins (RhoGAPs), respectively [13,14]. While RhoGEFs were initially given more attention, recent studies have revealed that RhoGAPs family members play critical roles in regulating tumor development and progression. Several RhoGAPs family members, including DLC1 (*ARHGAP7*), *ARHGAP22*, *ARHGAP24*, and *ARHGAP29*, were reported to play roles in renal cancer [15,16,17,18]. *ARHGAP11A* was highly expressed in metastatic RCC [19]. RhoGAPs family members were correlated with a tumor-promoting microenvironment in bladder cancer and gastric cancer [20,21]. However, it remains unclear whether RhoGAPs are involved in the immune microenvironment of ccRCC. Therefore, further investigation is needed to elucidate their roles and mechanisms in ccRCC immunity.

In this study, we found that *ARHGAP11A* served as an independent prognostic biomarker for ccRCC. ARHGAP11A promoted ccRCC cell proliferation and migration by contributing to the suppressive tumor immune microenvironment (TIME). A high *ARHGAP11A* level was correlated with infiltration of immunosuppressive cells, activation of immunosuppressive pathways, and expression of inhibitory IC. *ARHGAP11A* also promoted T cell exhaustion and induced immune escape. Renal tumors with low *ARHGAP11A* level responded to ICIs therapy. Our findings imply that the abnormal upregulation of ARHGAP11A promotes ccRCC development by forming an immunosuppressive microenvironment. *ARHGAP11A* level may also be a predictive biomarker for the response to ICIs therapy in ccRCC patients.

## 2. Results

### 2.1. ARHGAP11A Is an Independent Prognostic Biomarker for ccRCC

**Figure 1:**
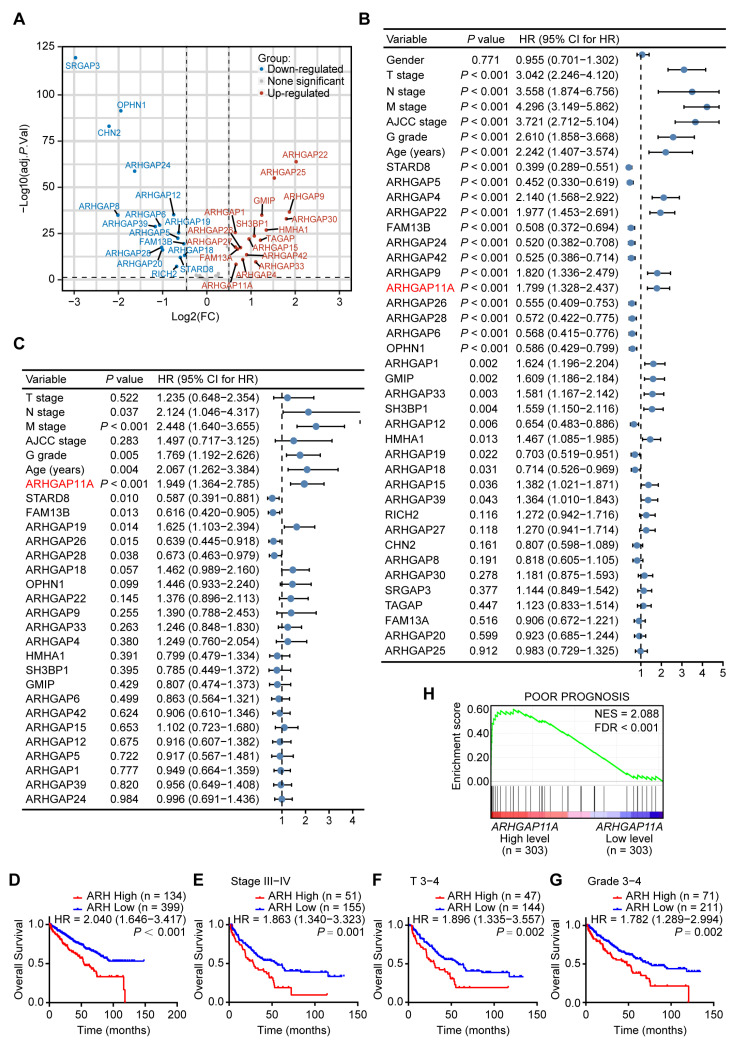
*ARHGAP11A* is a potential independent prognostic biomarker for ccRCC. (**A**) The volcano plot of 47 ARHGAPs genes in ccRCC samples (filtered criteria: |log_2_FC| > 0.5, adjusted *p*-value < 0.05). (**B**,**C**) Univariate (**B**) and multivariate (**C**) Cox regression analyses of RhoGAPs and patient overall survival in ccRCC, the gene in red is the target gene in this study. (**D**) The Kaplan–Meier (KM) curves of overall survival for ccRCC patients based on TCGA_KIRC data. (**E**–**G**) KM curves of overall survival for advanced ccRCC patients based on TCGA_KIRC data. (**H**) Enrichment plot of gene expression signature for poor prognosis (VANTVEER_BREAST_CANCER_POOR_PROGNOSIS) obtained by GSEA. The ccRCC samples from the TCGA_KIRC database were divided into high and low groups according to the quartile (**D**–**G**) or median value (**H**) of *ARHGAP11A* RNA-seq quantification results.

To explore which RhoGAPs family member played a critical role in ccRCC, we firstly analyzed the differential expression of 47 RhoGAPs based on the TCGA_KIRC dataset. Thirty-three RhoGAPs were found to be differentially expressed between ccRCC tissues and adjacent normal tissues (Figure 1A and Figure S1; |log_2_FC| > 0.5, adjusted *p*-value < 0.05). Then, univariate and multivariate Cox regression analyses were used to evaluate the prognostic value of these 33 differentially expressed genes (DEGs) in ccRCC. *ARHGAP11A* was found to be the only independent risk factor for ccRCC (Figure 1B,C, univariate analysis: hazard ratio (HR) 1.799, 95% confidence interval (CI) 1.328–2.437; multivariate analysis: HR 1.949, 95% CI 1.364–2.785). These results suggested that high expression of *ARHGAP11A* elevated mortality risk for ccRCC patients, and the mortality rate was 1.949 times higher than those with low expression of *ARHGAP11A*.

The association between *ARHGAP11A* level and survival rates of ccRCC patients was further analyzed. High *ARHGAP11A* level was correlated with shorter overall survival (OS) for ccRCC patients (HR 2.040, 95% CI 1.646–3.417), especially for patients with higher AJCC stage (HR 1.863, 95% CI 1.340–3.323), T stage (HR 1.896, 95% CI 1.335–3.557), and Fuhrman grade (HR 1.782, 95% CI 1.289–2.994). These results further suggested high expression of *ARHGAP11A* elevated mortality risk for ccRCC patients, and the mortality rate was 1.782–2.040 times higher than those with low expression of *ARHGAP11A* (Figure 1D–G). In addition, the poor prognosis gene set was enriched in the *ARHGAP11A* high expression group, suggesting that high *ARHGAP11A* level was positively correlated with poor prognosis of ccRCC patients (Figure 1H). These results suggested that *ARHGAP11A* is a potential independent prognostic marker for ccRCC. Therefore, we chose *ARHGAP11A* for further study.

### 2.2. High ARHGAP11A mRNA Level Is Positively Associated with the Malignancy of ccRCC Patients and Is Maintained by RNA Stabilizer IGF2BP3

**Figure 2:**
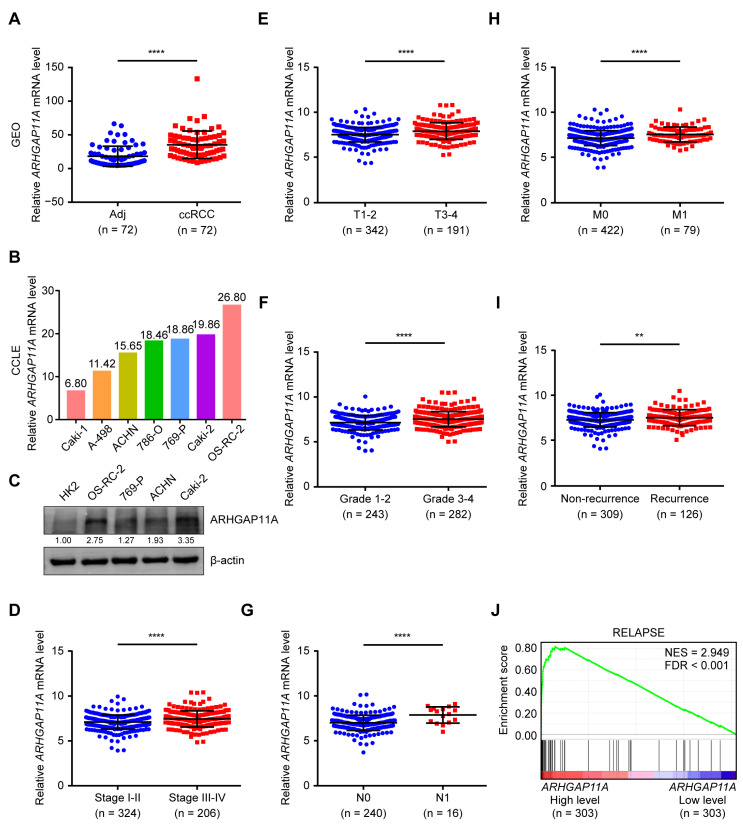
High *ARHGAP11A* level is positively correlated with the malignancy of ccRCC patients. (**A**,**B**) *ARHGAP11A* mRNA level was upregulated in both ccRCC tissues compared with adjacent normal tissues (**A**, GEO GSE53757 data) and RCC cells compared with immortalized renal epithelial HK2 cells (**B**, CCLE data). (**C**) WB verified that *ARHGAP11A* protein level was upregulated in RCC cells compared with HK2 cells. β-actin was used as a loading control. (**D**–**F**) *ARHGAP11A* mRNA level was gradually upregulated as AJCC stage (**D**), T stage (**E**), and Fuhrman grade (**F**) progressed. (**G**–**I**) *ARHGAP11A* mRNA level was positively correlated with lymph node metastasis (**G**), distant metastasis (**H**), and recurrence (**I**) in ccRCC patients. (**J**) Enrichment plot of gene expression signature for relapse (KAUFFMANN_MELANOMA_RELAPSE_UP) obtained by GSEA according to *ARHGAP11A* mRNA levels. The ccRCC samples from the TCGA_KIRC database were divided into high and low *ARHGAP11A* expression groups according to the median value of *ARHGAP11A* RNA-seq quantification results. ** *p* < 0.01, **** *p* < 0.0001.

Analysis results for the GEO (GSE53757) and CCLE databases verified that *ARHGAP11A* mRNA levels were upregulated in both RCC patient tumor samples and RCC cells (Figure 2A,B). Western blotting (WB) results revealed that *ARHGAP11A* protein level was also upregulated in RCC cells (Figure 2C). Further analytical results showed that *ARHGAP11A* mRNA level was gradually increased with the progression of AJCC stage, T stage, and Fuhrman grade (Figure 2D–F). Moreover, high *ARHGAP11A* level was found to be closely associated with metastasis and relapse (Figure 2G–J). These findings revealed that a high level of *ARHGAP11A* was positively correlated with the malignancy of ccRCC patients.

**Figure 3:**
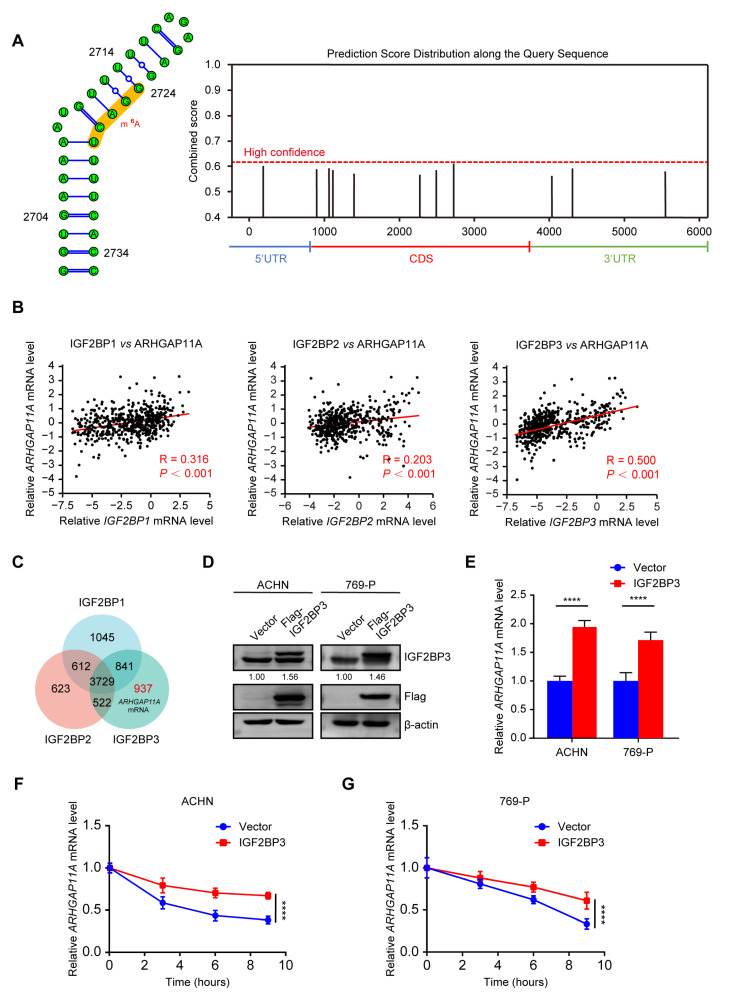
High *ARHGAP11A* mRNA level is stabilized by IGF2BP3. (**A**) The m6A modification site of *ARHGAP11A* mRNA predicted by the Sequence-Based Predictor of RNA Adenosine Methylation Sites (SRAMP) website tool. (**B**) The expression level correlations between *ARHGAP11A* and IGF2BP1/2/3 based on TCGA_KIRC data. (**C**) Venn diagram of RIP-Seq results GSE90639 for IGF2BP1–3. (**D**) WB verified IGF2BP3 was overexpressed in RCC cells ACHN and 769-P. (**E**) IGF2BP3 overexpression upregulated *ARHGAP11A* mRNA levels. (**F**,**G**) *ARHGAP11A* mRNA level was stabilized by IGF2BP3 overexpression. Cells overexpressing IGF2BP3 were treated with Act D in a time course. mRNA levels were determined by RT-qPCR (**E**–**G**). **** *p* < 0.0001.

Due to the important role of *ARHGAP11A* level in ccRCC malignancy, we further investigated the mechanism underlying the upregulation of *ARHGAP11A* mRNA level. m6A is the most important mRNA modification, as it can affect mRNA stability and degradation, ultimately leading to an increase in the target mRNA level [22]. *ARHGAP11A* mRNA had abundant m6A modification sites, mainly in the CDS region (Figure 3A). This indicated that *ARHGAP11A* mRNA level might be regulated by m6A modification. Among m6A readers, only the insulin-like growth factor 2 mRNA-binding proteins (IGF2BPs; including IGF2BP1/2/3) could increase mRNA stability. Hence, we first analyzed the expression level correlation between *ARHGAP11A* and each member of the IGF2BPs. *ARHGAP11A* level was positively correlated with all members of the IGF2BPs (Figure 3B, R = 0.316, 0.203, 0.500 for IGF2BP1–3, respectively, all *p* < 0.001), with the highest correlation for IGF2BP3. Simultaneously, according to RNA immunoprecipitation and deep sequencing (RIP-seq) data GSE90639, *ARHGAP11A* mRNA was only present in the precipitation of IGF2BP3; i.e., only IGF2BP3 could bind to *ARHGAP11A* mRNA (Figure 3C). Hence, we speculated that IGF2BP3 might increase *ARHGAP11A* mRNA level.

Cell experiments were performed to verify the enhancing effect of *IGF2BP3* on *ARHGAP11A* mRNA. When IGF2BP3 was overexpressed in ACHN and 769-P cells, *ARHGAP11A* mRNA level was increased and its stability was enhanced (Figure 3D–G). These results revealed that the upregulation of *ARHGAP11A* mRNA level in RCC cells was maintained by RNA stabilizer IGF2BP3.

### 2.3. ARHGAP11A Promotes Renal Cancer Cell Proliferation and Migration

**Figure 4:**
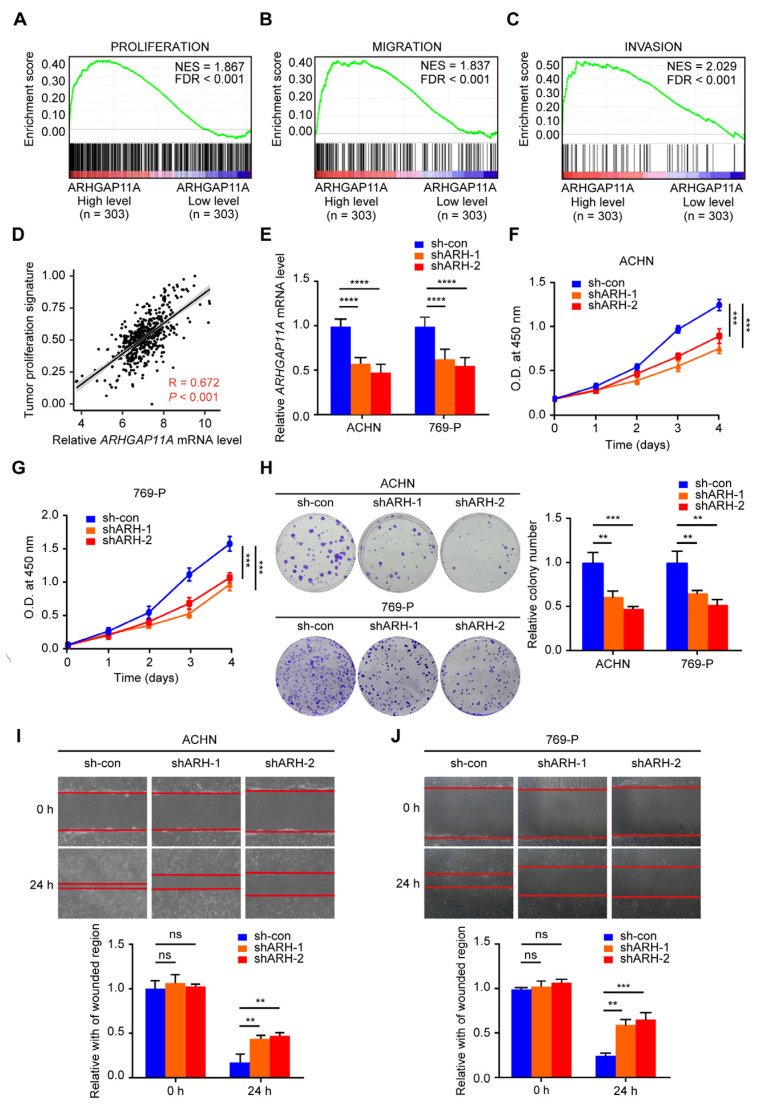
*ARHGAP11A* promotes renal cancer cell proliferation and migration. (**A**–**C**) Enrichment plots of gene expression signature for proliferation (**A**, CELL_PROLIFERATION_GO_0008283), migration (**B**, GOBP_MYELOID_LEUKOCYTE_MIGRATION), and invasion (**C**, PUIFFE_INVASION_INHIBITED_BY_ASCITES_UP) obtained by GSEA according to *ARHGAP11A* mRNA levels. The ccRCC samples from the TCGA_KIRC database were divided into high and low *ARHGAP11A* expression groups according to the median value of *ARHGAP11A* RNA-seq quantification results. (**D**) The correlation between *ARHGAP11A* level and the proliferation pathway by ssGSEA. (**E**) RT-qPCR results showed that *ARHGAP11A* was knocked down in RCC cell lines following transfection with ARHGAP11 knockdown plasmid compared with control vector. (**F**,**G**) *ARHGAP11A* knockdown suppressed RCC cell proliferation by CCK8 viability assay. (**H**) *ARHGAP11A* knockdown suppressed RCC cell colony formation by plate colony formation assay. (**I**,**J**) *ARHGAP11A* knockdown suppressed RCC cell migration by wound healing assay. ** *p* < 0.01, *** *p* < 0.001, **** *p* < 0.0001, ns—not significant.

The high level of *ARHGAP11A* in ccRCC tissues suggested that *ARHGAP11A* might play a tumor-promoting role in ccRCC. To verify this possibility, we first analyzed the correlation between *ARHGAP11A* level and ccRCC phenotype by GSEA. The results showed that high *ARHGAP11A* level was positively correlated with ccRCC cell proliferation, migration, and invasion (Figure 4A–C). We also found a highly positive correlation between *ARHGAP11A* expression and tumor proliferation signature score in each ccRCC sample by ssGSEA (Figure 4D, R = 0.670, *p* < 0.001). These results suggested that the high expression of AHGAP11A might promote the occurrence and development of ccRCC.

We subsequently knocked down *ARHGAP11A* and observed the cell phenotypes. *ARHGAP11A* knockdown suppressed the proliferation, colony formation, and migration of ACHN and 769-P cells (Figure 4E–J). These results revealed that *ARHGAP11A* exerted a tumor-promoting effect in RCC.

### 2.4. ARHGAP11A Is Associated with Immune Response

**Figure 5:**
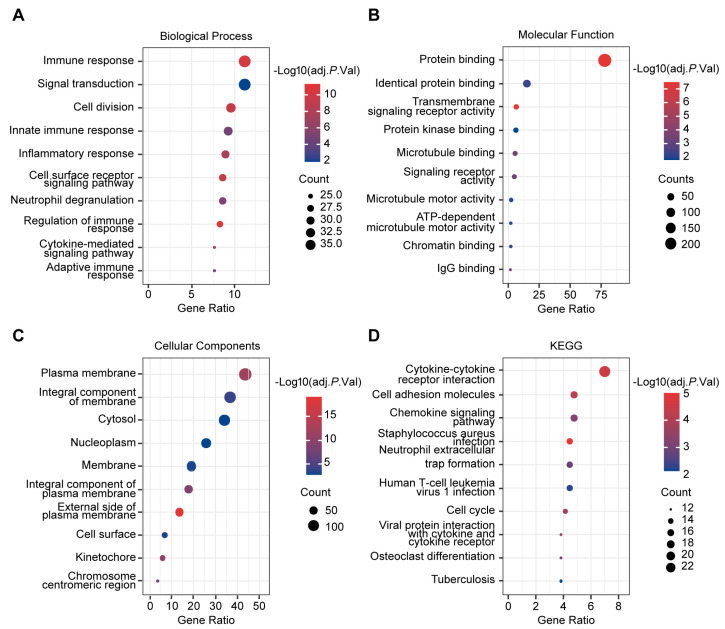
*ARHGAP11A* is associated with immune response. (**A**–**C**) Bubble chart of biological processes (**A**), molecular functions (**B**) and cellular components (**C**) according to GO analyses of *ARHGAP11A*-related DEGs. (**D**) KEGG pathway enrichment analysis of *ARHGAP11A*-related DEGs.

We next explored the mechanism by which *ARHGAP11A* promoted ccRCC occurrence and development. Gene Ontology (GO) and Kyoto Encyclopedia of Genes and Genomes (KEGG) pathway enrichment analyses were performed by *ARHGAP11A*-related DEGs. For biological processes, these genes were primarily involved in multiple immune responses and signal transduction (Figure 5A). Their molecular functions included protein binding, identical protein binding, and signaling receptor activity (Figure 5B). For cellular components, they were correlated with the plasma membrane, the integral component of the membrane, and cytosol (Figure 5C). KEGG results also showed that they were mainly enriched in the immune-related pathway, such as cytokine-cytokine receptor interaction, chemokine signaling pathway, neutrophil extracellular trap formation, etc. (Figure 5D). These results suggested that *ARHGAP11A* might promote ccRCC development via its role in immune response.

### 2.5. High ARHGAP11A Level Contributes to the Suppressive TIME in ccRCC

**Figure 6:**
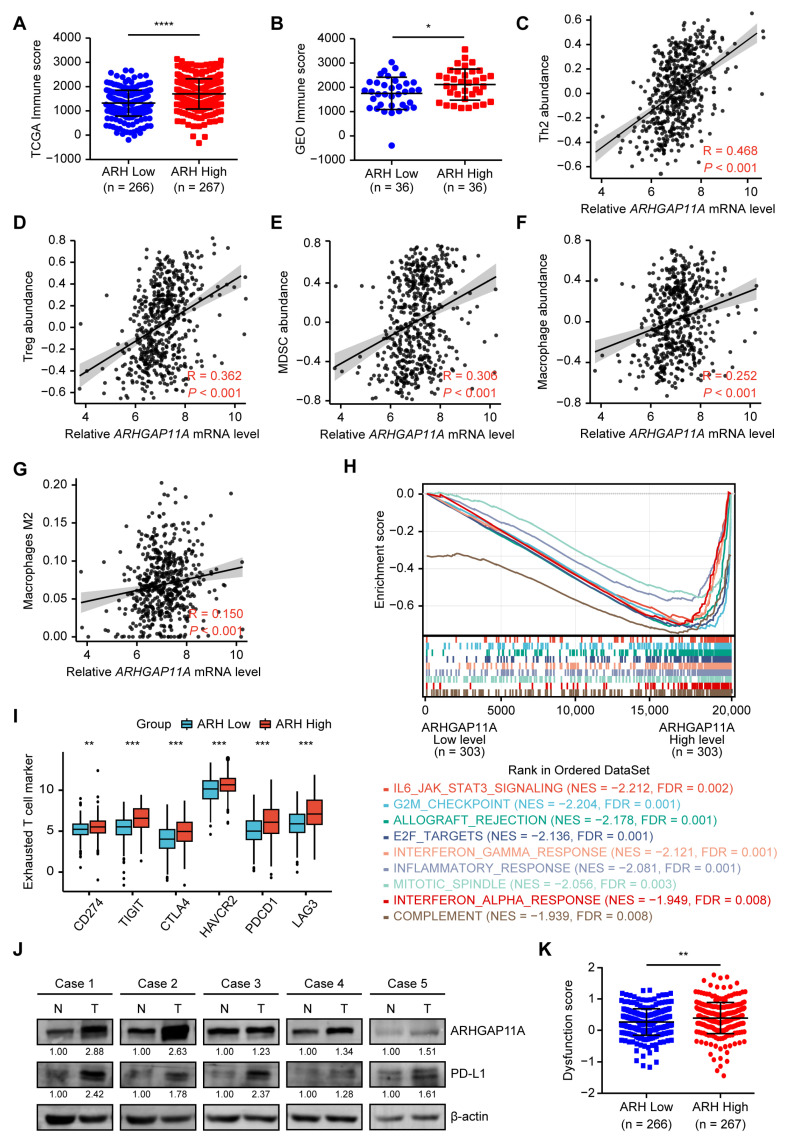
High *ARHGAP11A* level contributes to suppressive TIME in ccRCC. (**A**,**B**) Comparison of immune score between the high- and low-*ARHGAP11A* groups based on the ESTIMATE algorithm for TCGA_KIRC data (**A**) and GSE53757 data (**B**). (**C**–**F**) *ARHGAP11A* level was positively correlated with abundance of Th2 (**C**), Treg (**D**), MDSC (**E**), and macrophage (**F**) cells based on the KIRC TISIDB database. (**G**) *ARHGAP11A* level was positively correlated with abundance of M2 macrophages based on the CIBERSORT algorithm from the TCGA_KIRC database. (**H**) Enrichment plot of *ARHGAP11A* level for hallmark gene sets. The ccRCC samples from the TCGA_KIRC database were divided into high and low *ARHGAP11A* expression groups according to the median value of *ARHGAP11A* RNA-seq quantification results. (**I**) Exhausted T cell markers were upregulated in the *ARHGAP11A* high-level group. (**J**) *ARHGAP11A* and PD-L1 protein levels were concurrently upregulated in ccRCC tissues compared with paired normal tissues. Protein levels were detected by WB assay. (**K**) Dysfunction score for T cells was upregulated in the *ARHGAP11A* high-level group. * *p* < 0.05, ** *p* < 0.01, *** *p* < 0.001, **** *p* < 0.0001.

To elucidate the relationships between *ARHGAP11A* level and immune response in RCC, the immune score was analyzed by using the ESTIMATE algorithm based on TCGA and GEO data. The results showed that the samples with high *ARHGAP11A* level had more immune cell infiltration in tumor tissues (Figure 6A,B). Further analysis from the TIMER database demonstrated a weak positive correlation for *ARHGAP11A* level and infiltration levels of CD8^+^ T cells and CD4^+^ T cells (R = 0.267, 0.283, respectively, *p* < 0.001) and a moderate positive correlation of *ARHGAP11A* level with infiltration levels of B cells, macrophages, neutrophil, and dendritic cells (Figure S2A**,** R = 0.377, 0.394, 0.490, 0.479, respectively, all *p* < 0.001). Normally, immune cells killed tumor cells. More immune cell infiltration would suppress tumor development. This seemed to be contradictory to the tumor-promoting role of *ARHGAP11A*.

Given that immune cells had diverse subtypes and different subtypes exerted immune-promoting or inhibiting roles, we explored the TILs subtypes to solve this seemingly contradictory phenomenon. TILs subtypes that might be regulated by *ARHGAP11A* were analyzed based on correlations between the abundance of 28 TIL subtypes and *ARHGAP11A* level from the TISIDB database (Figure S2B). *ARHGAP11A* level was positively correlated with the abundance of most TILs, especially T helper 2 (Th2) cells, Tregs, myeloid derived suppressor cells (MDSC) (Figure 6C–E, moderate correlation, R = 0.468, 0.362, 0.306, respectively, all *p* < 0.001), and macrophage (Figure 6F, weak correlation, R = 0.252, *p* < 0.001). Macrophage M2 polarization could promote tumor development [23]. Hence, we further validated the association of *ARHGAP11A* level with M1/2 macrophages in ccRCC by the CIBERSORT algorithm. The results showed a weak positive correlation of AHGAP11A level with macrophage M2 (R = 0.150, *p* < 0.001), but not macrophage M1 (Figure 6G and Figure S2C–E). Inability of TILs to mediate antitumor function was likely to be due to the immune inhibitory effects mediated by Th2, Treg, and myeloid cell types [8]. Our results suggested that high *ARHGAP11A* level might contribute to a suppressive TIME by immune inhibitory effects of Th2, Treg, MDSC, and M2 macrophage. Further analysis results of the TIMER database demonstrated that *ARHGAP11A* was positively associated with the levels of immunosuppressive cells markers (Table 1), revealing the contribution of *ARHGAP11A* to suppressive TIME by immunosuppressive cells.

The correlation analyses of *ARHGAP11A* level with all hallmark gene sets showed that high *ARHGAP11A* level was correlated with immune response-related pathways (Figure 6H and Figure S2F, IL6-JAK-STAT3 signaling and interferon gamma (IFNγ) response). These immune response-related pathways could induce tumor immune escape [24,25]. These results revealed that *ARHGAP11A* might enhance the infiltration of immunosuppressive cells through IL6-JAK-STAT3 signaling and IFNγ response.

Both the infiltration of immunosuppressive cells and activation of IL6-JAK-STAT3 signaling/IFNγ response could affect the state of T cells [26]. Our results revealed that markers of exhausted T cells were highly expressed in the *ARHGAP11A* high-level group (Figure 6I). We selected the most critical marker for exhausted T cell, CD274 (PD-L1), to examine its expression in clinical specimens. The results showed a concurrent upregulation of *ARHGAP11A* and PD-L1 protein levels in ccRCC tissues (Figure 6J). These results indicated that when *ARHGAP11A* was upregulated, a large number of exhausted T cells existed in the tumor microenvironment. Moreover, the Tumor Immune Dysfunction and Exclusion (TIDE) analysis further showed that the group with a high level of *ARHGAP11A* had a higher dysfunction score, but not exclusion score for T cells (Figure 6K and Figure S2F). This indicated that patients with high *ARHGAP11A* level and immune infiltration tended to have a prominent T cell dysfunction signature, which impaired the ability of cytotoxic T cells to eliminate cancer cells.

### 2.6. Renal Tumors with Low ARHGAP11A Level Are Sensitive to ICIs Treatment

**Figure 7:**
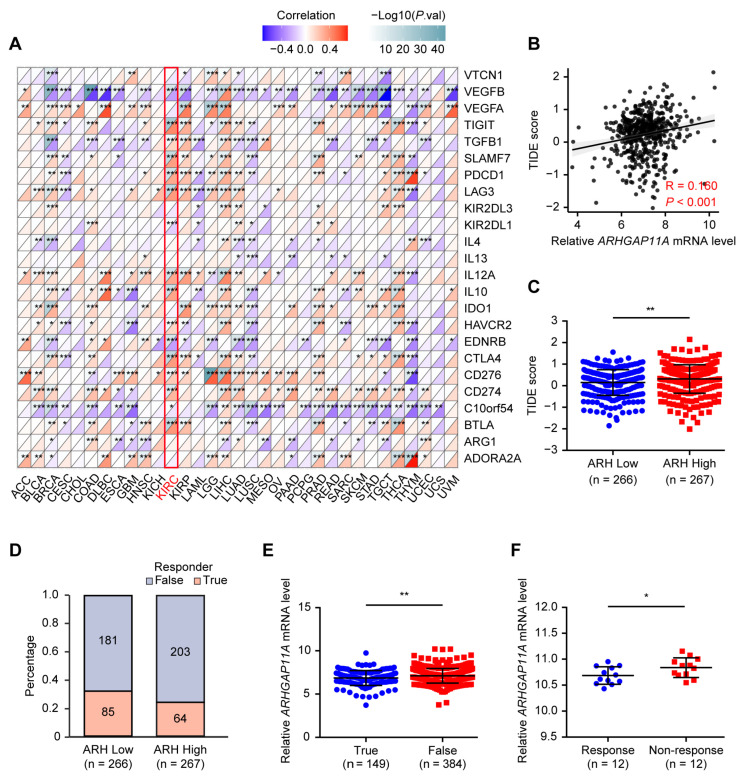
Renal tumors with low *ARHGAP11A* level are sensitive to ICIs therapy. (**A**) Correlation of RNA levels with inhibitory ICs expression in pan-cancer, red represented positive correlation, blue represented negative correlation. The darker the color, the stronger the correlation. (**B**) High *ARHGAP11A* level was positively associated with TIDE scores. (**C**) ccRCC patients with high *ARHGAP11A* level have a higher TIDE score. (**D**) The distribution of “True” or “False” responder in the high and low *ARHGAP11A* groups through the TIDE algorithm. (**E**) “TRUE-responder” group had a lower *ARHGAP11A* level. (**F**) *ARHGAP11A* expression in renal cancer-bearing mice responding or not responding to anti-PD-L1 and anti-CTLA-4 therapy. * *p* < 0.05, ** *p* < 0.01, *** *p* < 0.001.

Currently, ICIs therapy is an important strategy for advanced ccRCC [27]. We found that high *ARHGAP11A* level was positively associated with expression of inhibitory ICs in ccRCC patients (Figure 7A), suggesting a potential role of *ARHGAP11A* level in immunotherapy effectiveness prediction. Moreover, higher TIDE score could predict poorer response to ICIs treatment [28]. High *ARHGAP11A* level was positively correlated with higher TIDE score (Figure 7B,C, weak correlation, R = 0.160, *p* < 0.001), suggesting that patients with low *ARHGAP11A* level might respond to ICIs therapy. Further analyses showed that a higher proportion of ccRCC patients responded to ICIs therapy in the *ARHGAP11A* low-level group, and the “TRUE-responder” group had a lower *ARHGAP11A* level (Figure 7D,E). In addition, by analyzing gene expression profiles from renal cancer-bearing mice treated with an anti-PD-L1 and anti-CTLA-4 combination therapy, we confirmed that renal cancer-bearing mice responding to immunotherapy had a lower level of *ARHGAP11A* (Figure 7F). Therefore, ICIs treatment might be effective for renal tumors with low *ARHGAP11A* level.

## 3. Discussion

In this study, we found that high *ARHGAP11A* level was associated with shorter OS for ccRCC patients, especially for advanced ccRCC patients. We also found that upregulation of IGF2BP3 was a new mechanism for increasing the *ARHGAP11A* mRNA level in ccRCC. *ARHGAP11A* promoted ccRCC occurrence and development by contributing to a suppressive TIME. High *ARHGAP11A* level was associated with the infiltration of immunosuppressive cells, activation of IL6-JAK-STAT3 signaling and IFNγ response, and expression of inhibitory ICs. Renal tumors with low *ARHGAP11A* level were more likely to respond to ICIs therapy. These results provide a theoretical basis for *ARHGAP11A* as a novel prognostic marker, therapeutic target, and predictive marker for ICIs therapy in ccRCC.

This study newly found that *ARHGAP11A* promoted ccRCC development by inducing a suppressive TIME. In previous studies, *ARHGAP11A* was found to promote the development of basal breast cancer, colon cancer, hepatoma, and gastric cancer by accelerating cell transition from the G1 to S phase, inactivating Rac1B or regulating the TPM1-mediated actin filament stability [29,30,31,32]. While in gliomas, *ARHGAP11A* played a tumor-suppressing role by inducing cell-cycle arrest and apoptosis [33]. Surprisingly, high *ARHGAP11A* level was also reported to correlate with better prognosis in gastric cancer patients due to the detrimental effects on the suppressive TIME [20], which is inconsistent with its gastric cancer-promoting role. In all, it seemed that *ARHGAP11A* played diverse roles in different tumors via differential mechanisms.

Other members of RhoGAPs also played a tumor-promoting or -suppressing role via mechanisms completely different from *ARHGAP11A*. *ARHGAP5*, *ARHGAP17* and *ARHGAP24* promoted bladder cancer progression by establishing a tumor-promoting microenvironment or cellular mechanical property-mediated cell motility [21]. *ARHGAP10* inhibited the proliferation and metastasis of CRC cells via blocking the activity of the RhoA/AKT signaling pathway [34]. In gastric cancer, the fusion of *ARHGAP26* and the claudin-18 gene (*CLDN18*) led to the translation of an abnormal fusion protein, which promoted the development of gastric cancer [35]. In all, different members of RhoGAPs exerted tumor-promoting or -suppressing roles via different mechanisms.

There were two distinct immune escape mechanisms in TIME: a high level of infiltration by dysfunctional cytotoxic T cells and T cells exclusion from infiltrating tumors by immunosuppressive factors, which can be resolved by the TIDE score as immune dysfunction score and exclusion score. Renal cancer operates immune escape more through T cell dysfunction [28]. Immunoregulatory cell populations (Treg and myeloid cells), soluble factors, and environmental factors (IFN, IL10, IL6) and cell surface inhibitory receptors (PD-1, PD-L1, CTLA-4) were responsible for T cell exhaustion [8,9,26]. Myeloid cells can be enhanced by Th2 cells, which also promoted the immunosuppressive state of renal cancer [36]. IL-6/JAK/STAT3 signaling upregulated PD-L1 expression, while STAT3 positively regulated Treg cells and MDSC populations [25,37]. IFNγ enhanced PD-L1 and IDO expression in various tumors, which killed T cells and led to tumor immune escape [24]. In our work, we proved that *ARHGAP11A* was positively associated with infiltration of Treg, MDSC, and M2 macrophages cells. *ARHGAP11A* and its related proteins were involved in IL-6/JAK/STAT3 signaling and IFNγ response pathways. Immune cell markers of exhausted T cells were highly expressed in the *ARHGAP11A* high-level group. Therefore, *ARHGAP11A* might induce immune escape through exhausted T cells and participate in the formation of the suppressive TIME. Most RhoGAPs were positively correlated with infiltration of immune cells (Figure S3), suggesting that RhoGAPs might play a role in regulating ccRCC suppressive TIME.

Tumor immunotherapy had developed rapidly, and ICIs therapy killed tumor cells by regulating T cell activity. Several studies already reported the application of ICIs therapy in ccRCC patients [27]. Nivolumab (anti-PD-1 antibody) plus ipilimumab (anti-CTLA-4 antibody) had become the first-line treatment strategy for intermediate and poor-risk ccRCC patients [38]. TIDE score was the best predictor for anti-PD-1 and anti-CTLA-4 therapies. A higher tumor TIDE prediction score was associated not only with poorer response to ICIs therapy, but also with poorer patient survival under anti-PD1 and anti-CTLA-4 therapies [28]. Furthermore, the high levels of PD-1, PD-L1, CTLA-4, LAG-3, SIGLEC15, and TIGIT were associated with poor prognosis and immunosuppression of the tumor microenvironment in ccRCC [39,40,41]. Our results validated that the high *ARHGAP11A* group had a higher level of inhibitory ICs and TIDE score. In renal cancer-bearing mice, the *ARHGAP11A* low-level group responded more effectively to anti-PD-L1 plus anti-CTLA-4 immunotherapy. All these results suggested that ICIs treatment might be effective for ccRCC patients with low *ARHGAP11A* level. However, for ccRCC patients with high *ARHGAP11A* level, a combination of *ARHGAP11A* blockade and ICIs might obtain a better antitumor effect.

LncRNA and piRNA were reported to upregulate *ARHGAP11A* level in human hepatocellular carcinoma (HCC) and breast cancer [42,43]. This study newly found that the upregulation of RNA stabilizer IGF2BP3 led to the upregulated *ARHGAP11A* level in ccRCC. IGF2BP3 was highly expressed in ccRCC and acted as an m6A reader, exerting tumor-promoting effects by enhancing mRNA stability and translation [44]. However, whether the regulation of *ARHGAP11A* by IGF2BP3 was dependent on m6A modification needs further investigation.

This study has some limitations and drawbacks. Firstly, further validation from specimens collected at other centers is required to confirm *ARHGAP11A* as a prognostic biomarker. Secondly, because the work about TIME was mostly dependent on bioinformatic methodologies, more experimental validations are required to elucidate the role and detailed molecular mechanisms of *ARHGAP11A* in suppressive TIME. Finally, the availability of appropriate cohorts of ccRCC patients undergoing immunotherapy is limited. We hope our results can be further confirmed in ccRCC immunotherapy cohorts. Certainly, an available effective small molecule targeting *ARHGAP11A* will help to verify the effect of *ARHGAP11A* as an independent or combined therapeutic target for immunotherapy. In summary, *ARHGAP11A* could be a new promising immune-related prognostic marker and therapeutic target for ccRCC. Additionally, *ARHGAP11A* might act as a predictive marker for ICIs therapy in ccRCC.

## 4. Materials and Methods

### 4.1. Data Collection

The Cancer Genome Atlas kidney cancer database (TCGA_KIRC) RNA Seq v2 mRNA data (Synapse ID: syn2320105), including 534 tumor samples and 72 adjacent normal samples, was downloaded from https://www.synapse.org/#!Synapse:syn2320105 (accessed on 22 April 2022) and corresponding clinical information for ccRCC patients was from the cBioPortal database (TCGA-Firehose Legacy, https://www.cbioportal.org/study/summary?id=kirc_tcga, accessed on 22 April 2022). The Gene Expression Omnibus (GEO) GSE53757 microarray dataset, including 72 pairs of ccRCC tumors and adjacent normal samples, was downloaded from https://www.ncbi.nlm.nih.gov/geo/query/acc.cgi?acc=GSE53757 (accessed on 22 April 2022). For obtaining the Cancer Cell Line Encyclopedia mRNA expression dataset of kidney cancer cells, https://depmap.org/portal/download/all/ (accessed on 22 April 2022) was searched first, and then the “CCLE 2019” dataset was selected. Transcriptomic data of TCGA Pan-cancer cohort were downloaded from the UCSC Xena data portal (https://xenabrowser.net, accessed on 22 April 2022) [45] to explore the correlation of *ARHGAP11A* level with expression of ICs across 33 different cancer types.

### 4.2. Tissue Collection

The ccRCC and adjacent normal kidney tissues from the same patient were collected from nephrectomy specimens at the Affiliated Beijing Friendship Hospital, Capital Medical University. Five pairs of fresh samples were immediately frozen in liquid nitrogen and stored at −80 °C for use in WB analysis. All specimens were histologically confirmed by pathologists. The study was approved by the Research Ethics Board of Affiliated Beijing Friendship Hospital and was performed according to the World Medical Association Declaration of Helsinki. The patients were included after signing written informed consent. Prior to surgery, the patients had not received any therapies.

### 4.3. Gene Set Enrichment Analysis and Single-Sample GSEA

The correlation of *ARHGAP11A* level with phenotypes was analyzed by gene set enrichment analysis (GSEA) [46]. GSEA calculates a gene set enrichment score (ES) that estimates whether genes from a predefined gene set (obtained from the Molecular Signatures Database, MSigDB) were enriched in the *ARHGAP11A* high/low group or distributed randomly. Default settings were used. Thresholds for significance were determined by permutation analysis (1000 permutations). False discovery rate (FDR) was calculated. A gene set is considered significantly enriched when the FDR score is <0.05. The “GSVA” package (version 1.46.0), calculating the ssGSEA score for each sample with the ssGSEA method and Gaussian kernel density estimation, was used to analyze the correlation of *ARHGAP11A* level with the pathway score of the tumor proliferation signature in each tumor sample. The tumor proliferation signature was obtained from MsigDB [47].

### 4.4. Prediction of ARHGAP11A m6A Site and m6A Reader

The m6A sites of *ARHGAP11A* (NM_014783.6) were predicted by Sequence-Based Predictor of RNA Adenosine Methylation Sites (SRAMP, an online m6A site predictor), as previously described [48]. Correlation and binding RNA sequencing result analyses were used to identify the specific m6A reader of *ARHGAP11A*. Since the IGF2BP family was the only m6A reader enhancing target mRNA stability, we analyzed and visualized expression level correlations between *ARHGAP11A* and IGF2BP1-3 by “corrplot” (version 0.92) based on TCGA_KIRC data. RIP-sequencing data GSE90639, which used Flag beads to precipitate Flag-tagged IGF2BP1/2/3 overexpressed in HEK293T cells, and further identified the mRNA binding targets of IGF2BP1/2/3 by RNA sequencing, was downloaded from https://www.ncbi.nlm.nih.gov/geo/query/acc.cgi?acc=GSE90639 (accessed on 22 April 2022).

### 4.5. Cell Lines and Cell Culture

The human renal carcinoma cell lines ACHN, HK2, OS-RC-2, and 769-P were obtained from Beijing XieHe cell bank (Beijing, China). Caki-2 was obtained from Procell Biotech (Wuhan, China). ACHN, OS-RC-2 and 769-P were cultured in RPMI-1640 medium. HK2 was cultured in DMEM/F12 (1:1) medium. Caki-2 was cultured in McCoy’s 5A medium. All mediums were purchased from Gibco Laboratories (Grand Island, NY, USA). These mediums were supplemented with 10% fetal bovine serum (Hyclone, Logan, UT, USA) and 1% streptomycin/penicillin (Life Technologies, Grand Island, NY, USA). Cells were cultured at 37 °C and 5% CO_2_.

### 4.6. Cell Transfection

pcDNA3.1-IGF2BP3-3×flag overexpression plasmid (Cat# F117530) and pPLK/GFP *ARHGAP11A* knockdown plasmid (sh*ARHGAP11A*-1: GTATCAGTTCACATCGATA; sh*ARHGAP11A*-2: CCTTCTATTACACCTCAAGAA) were purchased from Youbio (Changsha, China) and Public Protein/Plasmid Library (Nanjing, China), respectively. Cells were transfected using Lipofectamine LTX (Invitrogen, Carlsbad, CA, USA) according to the manufacturer’s instructions. For stable transfection, cells were transfected with the plasmid or control vector following the protocol and screened with G418 (800 µg/mL, Amresco, Solon, OH, USA) or puromycin (0.3 µg/mL, Sigma-Aldrich, St. Louis, MO, USA). Stably transfected cell pools were maintained and passaged in culture medium with G418 (400 µg/mL) or puromycin (0.15 µg/mL).

### 4.7. Western Blotting

WB was performed as previously described [49]. Both anti-IGF2BP3 (Cat# A4444) and anti-Flag (Cat# AE063) antibodies were purchased from Abclonal (Wuhan, China). Anti-*ARHGAP11A* (Cat# PA5-101840), anti-PD-L1 (Cat# M033179), and anti-β-actin (Cat# AC006) antibodies were purchased from Invitrogen, Abmart (Shanghai, China), and Sigma-Aldrich, respectively. The protein level was normalized with β-actin.

### 4.8. RNA Extraction and RT-qPCR Analysis

Total RNA was extracted from cultured cells by using Trizol reagent (TaKaRa, Dalian, China) and converted into cDNAs by using the Primer-Script one step RT-PCR kit (RiboBio, Guangzhou, China), following the manufacturer’s instruction. The cDNA template was amplified by real-time RT-PCR using the SYBR Premix Dimer Eraser kit (TaKaRa). Gene expression in each sample was normalized to GAPDH. Primers for reverse transcription and amplification of *ARHGAP11A* and GAPDH were synthesized by RiboBio. The specific primer sequences used in RT-qPCR were as follows: *ARHGAP11A*, 5′-GCAGGTGTGCCAAGGCGAAGT-3′ and 5′-TGCAAGTCGCCAACCAACACTTT CA-3′; GAPDH, 5′-CGGAGTCAACGGATTTGGTCGTAT-3′, and 5′-AGCCTTCTCCATGGTGGT GAAGAC-3′. Real-time PCR reactions were performed in triplicate by using an ABI7500 system (Applied Biosystems, Carlsbad, CA, USA). Relative expression level was calculated using the 2^−ΔΔCt^ method.

### 4.9. mRNA Stability Assay

ACHN and 769-P cells, stably expressing IGF2BP3 and control cells, were treated with 5 µg/mL actinomycin D (Act D, Sigma-Aldrich) to block transcription for 0, 3, 6, and 9 h. RNA was isolated from cells, and *ARHGAP11A* mRNA level was detected by RT-qPCR assay and normalized with GAPDH mRNA.

### 4.10. Cell Phenotype Assays

Cell Counting Kit-8 (Dojindo, Kumamoto, Japan), colony-forming experiment, and scratch assay were conducted to determine cell proliferation ability and cell migration ability, respectively, as described previously [50].

### 4.11. Analyses of Differentially Expressed Genes

DEGs from syn2320105 were calculated by applying the R package “limma” (version 3.54.2) with LogCPM as the normalization method [51]. The differential cutoff criteria for 47 RhoGAPs were |log_2_FC| > 0.5 and adjusted *p*-value < 0.05. DEGs of 47 RhoGAPs were visualized using volcano plots and heatmaps, plotted by using the “ggplot2” (version 3.4.1) and “ggpubr” (version 0.6.0) packages, respectively [52]. The cutoff criteria of DEGs between tumor and adjacent normal samples were |log_2_FC| > 2 and adjusted *p*-value < 0.05.

### 4.12. GO and KEGG Analyses

*ARHGAP11A*-related DEGs were used to perform GO and KEGG analyses. To obtain *ARHGAP11A*-related DEGs, coexpressing genes of *ARHGAP11A* were first downloaded from the coexpression module at https://www.cbioportal.org/ (accessed on 22 April 2022) (TCGA_KIRC database, TCGA, Firehose Legacy). Subsequently, these *ARHGAP11A* coexpressing genes with the cutoff criteria of *p*-value < 0.05 and |R| > 0.3 were overlapped with DEGs between ccRCC and adjacent normal tissues. The overlapping genes were selected as *ARHGAP11A*-related DEGs. GO and KEGG pathway enrichment analyses of *ARHGAP11A*-related DEGs were executed by the online analysis tools—Database for Annotation, Visualization, and Integrated Discovery (DAVID) (https://david.ncifcrf.gov/, accessed on 26 April 2022)—against the background of *Homo sapiens* [53].

### 4.13. Correlation Analyses of ARHGAP11A Level with the TME and Immunotherapy Response

Immune score was estimated using the ESTIMATE algorithm (version 1.0.13) (https://bioinformatics.mdanderson.org/estimate/, accessed on 26 April 2022) to assess the association between *ARHGAP11A* level and the degree of immune cell infiltration in ccRCC [54]. The correlation of *ARHGAP11A* level with abundance of immune cells and the expression of immune markers in ccRCC were analyzed based on the TIMER database (https://cistrome.shinyapps.io/timer/, accessed on 26 April 2022) [55]. Relations between the abundance of 28 TILs and *ARHGAP11A* level in ccRCC were analyzed by using the TISIDB database (http://cis.hku.hk/TISIDB/index.php, accessed on 26 April 2022) [56]. Relations between the abundance of M1/M2 macrophages and *ARHGAP11A* level were further analyzed by the CIBERSORT algorithm (http://cibersort.stanford.edu/, accessed on 26 April 2022) with 1000 permutations for significance analysis [57]. Differential expressions of exhausted T cell markers between the high and low *ARHGAP11A* level groups were visualized by applying the “ggplot2” (version 3.4.1) and “ggpubr” (version 0.6.0) packages [52]. Correlations of *ARHGAP11A* level with ICs expression were analyzed and visualized by using the “corrplot” (version 0.92) [58], “ggplot2” (version 3.4.1), and “ggpubr” (version 0.6.0) packages. The TIDE analysis was employed to predict the immunotherapy responses of patients (http://tide.dfci.harvard.edu, accessed on 26 April 2022) [28]. GSE117358, RNA-sequencing data for 24 tumor samples from kidney cancer Renca-bearing mice pretreated with anti-PD-L1, and anti-CTLA-4 immunotherapy (12 responders, 12 nonresponders), were downloaded from GEO (https://www.ncbi.nlm.nih.gov/geo/query/acc.cgi?acc=GSE117358, accessed on 26 April 2022).

### 4.14. Statistical Analysis

Statistical analyses were performed using R (4.2.3), Graphpad Prism 8 (Graphpad Software, San Diego, CA, USA), and IBM SPSS 23 (SPSS, Chicago, IL, USA). Univariate and multivariate Cox proportional hazard regression analyses were used to estimate the prognostic significance of *ARHGAP11A* in ccRCC with the “survival” (version 3.5-5) package [59]. Difference in OS between the two groups was compared by the KM method and log-rank test. The paired samples *t*-test and independent samples *t*-test were used to analyze the statistical significance of paired and unpaired samples within two groups, respectively. The significance among more than two groups and the proliferation curve result were analyzed with one-way analysis of variance (ANOVA) and repeated measures ANOVA, respectively, with least-significant difference (LSD) correction. The data were presented as mean ± SD. Correlation coefficients were obtained using the Spearman correlation analysis (R values with 0.1–0.3, 0.3–0.7, and >0.7 were defined as weak, moderate, or strong correlation, respectively). A *p* < 0.05 was deemed as statistically significant.

## Figures and Tables

**Table 1:** Correlations of *ARHGAP11A* with immune markers level in the TIMER database unadjusted or adjusted by purity.

Immune Cell	Biomarker	KIRC			
		None		Purity	
		Cor	*p*	Cor	*p*
Monocyte	CD86	0.464	***	0.472	***
	CD115	0.428	***	0.408	***
TAM	CD68	0.395	***	0.407	***
	IL10	0.419	***	0.404	***
M1 Macrophage	INOS	0.099	*	0.072	ns
	IRF5	0.288	***	0.303	***
	COX2	0.188	***	0.166	***
M2 Macrophage	CD163	0.467	***	0.460	***
	VSIG4	0.413	***	0.393	***
	MS4A4A	0.428	***	0.429	***
Neutrophils	CD66b	0.093	*	0.112	*
	CD11B	0.442	***	0.425	***
	CCR7	0.284	***	0.266	***
Natural killer cell	KIR2DL1	−0.004	ns	−0.030	ns
	KIR2DL3	0.024	ns	0.021	ns
	KIR3DL1	−0.015	ns	−0.001	ns
	KIR3DL2	−0.047	ns	−0.042	ns
	KIR3DL3	0.040	ns	0.042	ns
	KIR2DS4	−0.047	ns	−0.052	ns
Dendritic cell	HLA-DPB1	0.307	***	0.305	***
	HLA-DQB1	0.146	**	0.125	**
	HLA-DRA	0.379	***	0.392	***
	HLA-DPA1	0.380	***	0.390	***
	CD1C	0.150	***	0.134	**
	NRP1	0.245	***	0.224	***
	ITGAX	0.320	***	0.311	***
Th1	TBX21	0.176	***	0.162	***
	STAT4	0.326	***	0.314	***
	STAT1	0.579	***	0.590	***
	TNF-α	0.294	***	0.286	***
Th2	GATA3	0.090	*	0.085	ns
	STAT6	0.189	***	0.197	***
	STAT5A	0.372	***	0.350	***
Treg	FOXP3	0.358	***	0.356	***
	CCR8	0.457	***	0.471	***
	STAT5B	0.193	***	0.190	***
	TGFB1	0.271	***	0.222	***

Cor—R value of Spearman’s correlation; * *p* < 0.05, ** *p* < 0.01, ***p < 0.001, ns—no significance.

## Data Availability

Publicly available datasets were analyzed in this study. These data can be found at: https://www.synapse.org/#!Synapse:syn2320105 (accessed on 22 April 2022), https://www.cbioportal.org/study/summary?id=kirc_tcga (accessed on 22 April 2022), https://www.ncbi.nlm.nih.gov/geo/ (accessed on 22 April 2022), https://depmap.org/portal/download/all/ and https://xenabrowser.net (accessed on 22 April 2022).

## Supplementary Materials

The following supporting information can be downloaded at: https://www.mdpi.com/article/10.3390/ijms24097755/s1.
