# Supplementary material for: ARHGAP11A Is a Novel Prognostic and Predictive Biomarker Correlated with Immunosuppressive Microenvironment in Clear Cell Renal Cell Carcinoma
Source: Int J Mol Sci. 2023 Apr 24;24(9):7755. doi: 10.3390/ijms24097755 (PMC10178328; doi:10.3390/ijms24097755)
Supplement: Supplementary file 1 [file ijms-24-07755-s001.zip › ijms-2273366-Supplementary Materials.pdf]

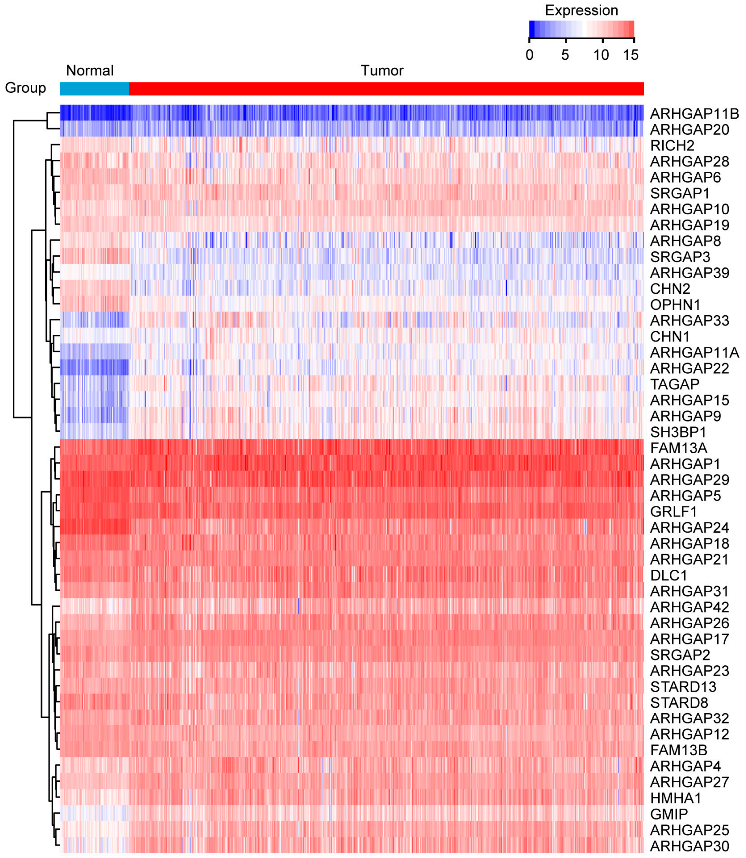

Supplementary Figure S1. The heatmap of 47 RhoGAPs genes in normal and ccRCC samples.

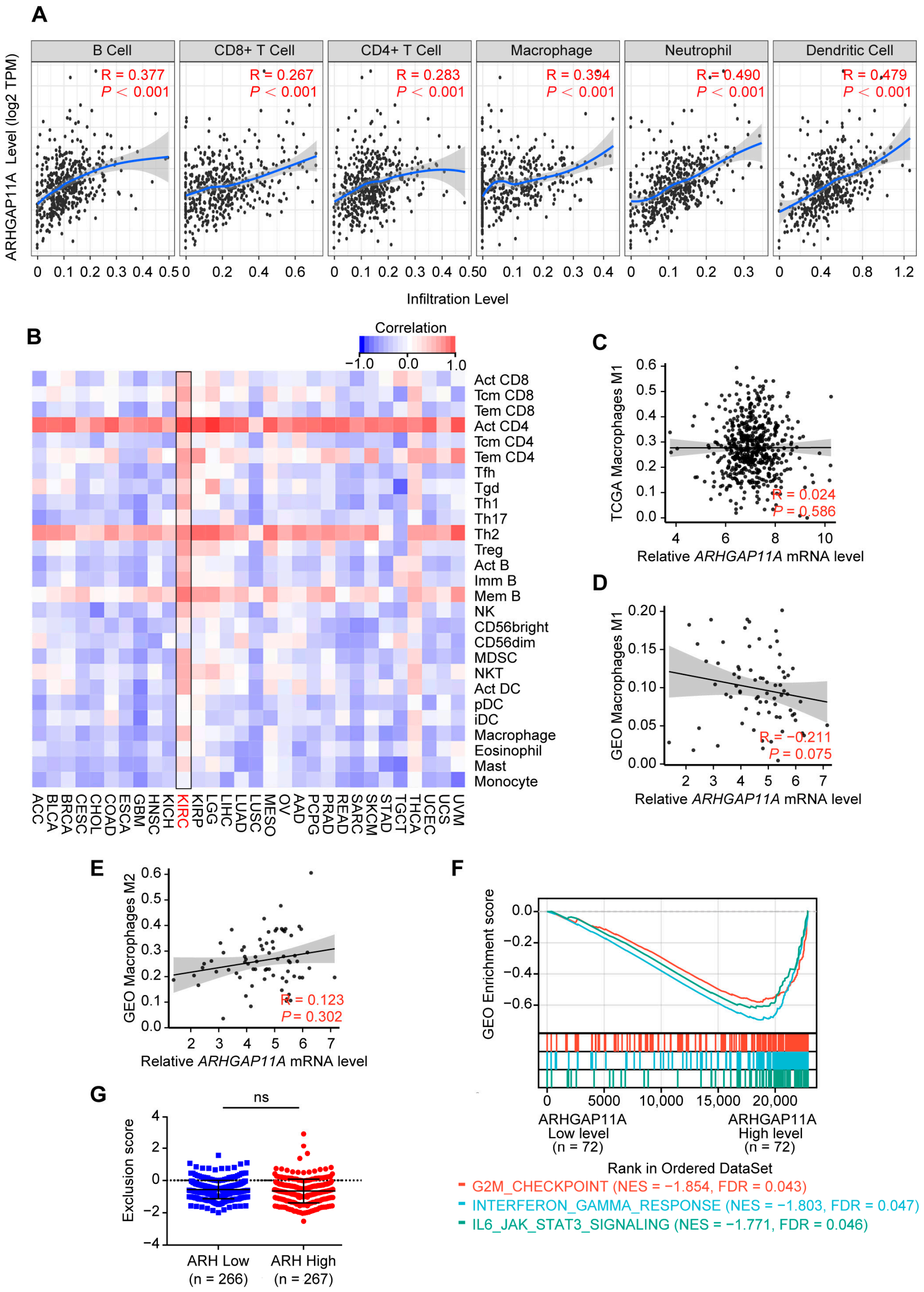

Supplementary Figure S2. The relationship between *ARHGAP11A* and TIME score. (**A**) Correlations between *ARHGAP11A* level and 8 types of TILs abundance based on TIMER database of KIRC. (**B**) Correlation between *ARHGAP11A* level and 28 types of TILs abundance based on TISIDB database of pan tumor. (**C-E**) Relations between *ARHGAP11A* level and abundance of M1/M2 macrophages from TCGA_KIRC and GEO GSE53757 database based on "CIBERSORT" algorithm. (**F**) Enrichment plot of *ARHGAP11A* level in hallmark gene sets. The ccRCC samples from GEO GSE53757 database were divided into high and low *ARHGAP11A* expression groups according to the median value of *ARHGAP11A* RNA-seq quantification results. (**G**) Differences in exclusion score between high- and low-*ARHGAP11A* group.

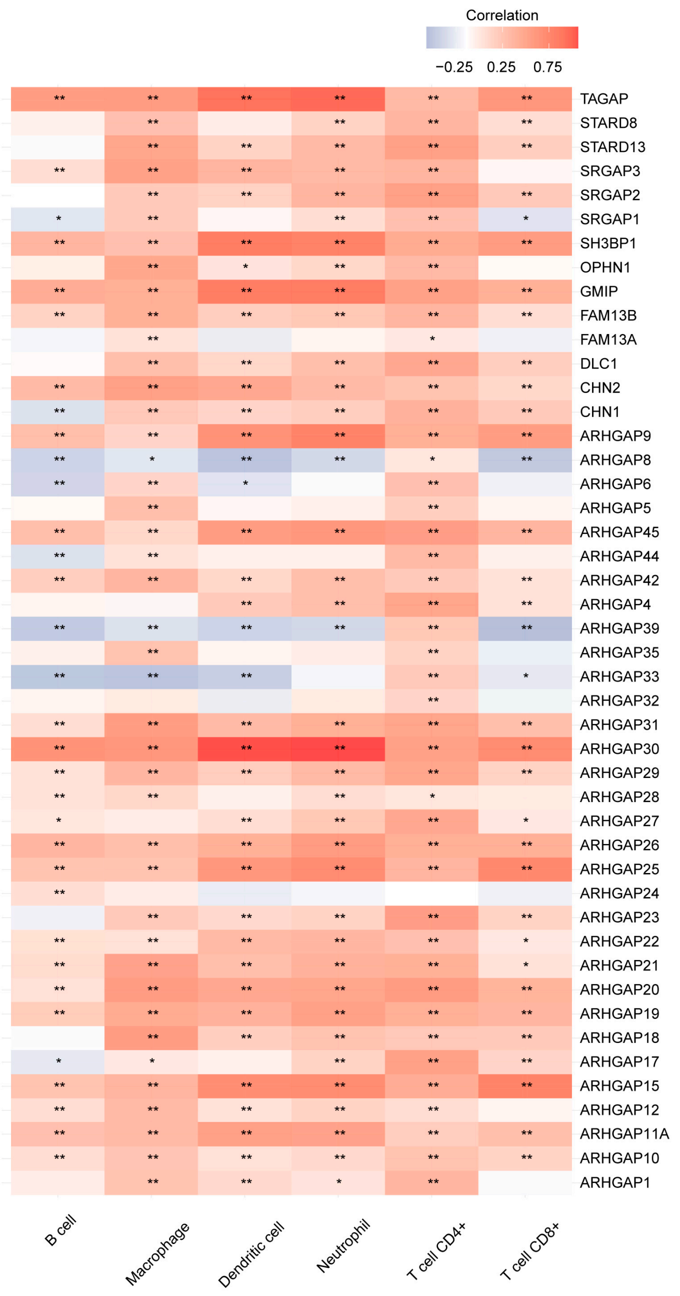

Supplementary Figure S3. Heatmap of correlations between expression levels of RhoGAPs and immune cell infiltration. The horizontal axis represents immune cell types, the vertical axis represents genes, red is positive correlation, blue is negative correlation. The darker color, the stronger correlation.
